# BH3-only protein expression determines hepatocellular carcinoma response to sorafenib-based treatment

**DOI:** 10.1038/s41419-021-04020-z

**Published:** 2021-07-26

**Authors:** Stephanie Busche, Katharina John, Franziska Wandrer, Florian W. R. Vondran, Ulrich Lehmann, Heiner Wedemeyer, Frank Essmann, Klaus Schulze-Osthoff, Heike Bantel

**Affiliations:** 1 Department of Gastroenterology, Hepatology and Endocrinology, Hannover Medical School, Hannover, Germany; 2 Department of Visceral and Transplantation Surgery, Hannover Medical School, Hannover, Germany; 3 German Centre for Infection Research (DZIF), partner site Hannover-Braunschweig, Hannover, Germany; 4 Department of Pathology, Hannover Medical School, Hannover, Germany; 5 Dr. Margarete-Fischer-Bosch Institute of Clinical Pharmacology, Stuttgart, Germany; 6 Interfaculty Institute of Biochemistry, University of Tübingen, Tübingen, Germany; 7 German Cancer Consortium (DKTK) and German Cancer Research Center (DKFZ), Heidelberg, Germany; 8 Cluster of Excellence iFIT (EXC 2180) “Image-Guided and Functionally Instructed Tumor Therapies”, University of Tübingen, Tübingen, Germany

**Keywords:** Cancer, Diseases

## Abstract

Hepatocellular carcinoma (HCC) represents a global health challenge with limited therapeutic options. Anti-angiogenic immune checkpoint inhibitor-based combination therapy has been introduced for progressed HCC, but improves survival only in a subset of HCC patients. Tyrosine-kinase inhibitors (TKI) such as sorafenib represent an alternative treatment option but have only modest efficacy. Using different HCC cell lines and HCC tissues from various patients reflecting HCC heterogeneity, we investigated whether the sorafenib response could be enhanced by combination with pro-apoptotic agents, such as TNF-related apoptosis-inducing ligand (TRAIL) or the BH3-mimetic ABT-737, which target the death receptor and mitochondrial pathway of apoptosis, respectively. We found that both agents could enhance sorafenib-induced cell death which was, however, dependent on specific BH3-only proteins. TRAIL augmented sorafenib-induced cell death only in NOXA-expressing HCC cells, whereas ABT-737 enhanced the sorafenib response also in NOXA-deficient cells. ABT-737, however, failed to augment sorafenib cytotoxicity in the absence of BIM, even when NOXA was strongly expressed. In the presence of NOXA, BIM-deficient HCC cells could be in turn strongly sensitized for cell death induction by the combination of sorafenib with TRAIL. Accordingly, HCC tissues sensitive to apoptosis induction by sorafenib and TRAIL revealed enhanced NOXA expression compared to HCC tissues resistant to this treatment combination. Thus, our results suggest that BH3-only protein expression determines the treatment response of HCC to different sorafenib-based drug combinations. Individual profiling of BH3-only protein expression might therefore assist patient stratification to certain TKI-based HCC therapies.

## Introduction

Hepatocellular carcinoma represents a serious global health problem because of its increasing incidence and the limited therapeutic options [1]. Among available treatment options for patients with advanced HCC are the tyrosine kinase inhibitors (TKI) sorafenib and lenvatinib that target VEGFR1-3, FGFR1-4, PDGFR, RET, and KIT. These TKIs achieve a modest treatment response but remain so far the most effective single-drug therapy for HCC [2, 3]. Recently, a combination therapy of the immune checkpoint inhibitor atezolizumab (anti-PDL1) and bevacizumab (anti-VEGF) has been approved as first-line therapy for advanced HCC. The progression-free survival could be increased by this combination therapy compared to sorafenib monotherapy [4]. It appears therefore important to combine anti-cancer drugs that target different signaling pathways to enhance the anti-tumor treatment efficacy [5]. In this respect, the combination of lenvatinib with the immune checkpoint inhibitor nivolumab (anti-PD1) is currently evaluated as first-line therapy in HCC (IMMUNIB trial, NCT03841201). Nevertheless, oncogenic activation of survival pathways can occur and contribute to treatment resistance of HCC. For instance, activation of the Wnt/β-catenin pathway, which is caused by mutations of e.g., *CTNNB1* (encoding for β-catenin) and found in 30−40% of HCCs, results in resistance to checkpoint inhibitors [6–8]. Therefore, to improve the survival of HCC patients, it is fundamental to understand the mechanisms of therapy resistance and to unravel further molecular targets, which enable the creation of an effective individual treatment strategy.

Tyrosine kinase inhibitors, such as sorafenib, inhibit proliferation and induce apoptosis in human cancer cells by inhibition of the Raf-MAPK pathway and of transcription/translation factors, such as NF-κB, STAT3, eIF2, eIF4e, which e.g., leads to the downregulation of anti-apoptotic Bcl-2 molecules [9–11]. Anti-apoptotic regulators are highly expressed in HCC and contribute to treatment resistance [12]. The application of drugs that counteract anti-apoptotic mechanisms therefore represents a promising treatment strategy for HCC.

Apoptosis induction can be mediated by the extrinsic or intrinsic death pathway [12, 13]. The extrinsic pathway involves the engagement of death receptors by ligands of the TNF superfamily, such as CD95L or TNF-related apoptosis-inducing ligand (TRAIL). The binding of these ligands to their cognate receptors results in the recruitment of the initiator caspase-8/-10 into the death-inducing signaling complex, wherein caspase-8/-10 becomes activated [14]. In type II cells, such as hepatocytes, the extrinsic pathway is amplified by caspase-8-mediated cleavage of the pro-apoptotic Bcl-2 molecule BID, which triggers together with BAX and BAK the release of pro-apoptotic mitochondrial factors, thereby inducing the activation of the intrinsic death pathway [13]. A key event of the intrinsic pathway is the mitochondrial release of cytochrome c, which enables the formation of the apoptosome and activation of the initiator caspase-9. Initiator caspases then activate downstream effector caspases, such as caspase-3 and -7, which cleave a variety of cellular substrates and subsequently trigger apoptosis [13]. The intrinsic pathway of apoptosis is tightly controlled by members of the Bcl-2 family, which are classified in pro-apoptotic and anti-apoptotic (e.g., BCL-2, BCL-X_L_, and MCL-1) proteins. Pro-apoptotic members are further divided into multidomain Bcl-2 proteins, such as BAX, BAK, and BOK, and proteins that harbor only a single BH3 domain (i.e., BAD, BID, BIM, PUMA, and NOXA) and are therefore called BH3-only proteins [14–17].

Increasing evidence suggests that BH3-only proteins determine the apoptotic outcome, which has led to the development of small-molecule inhibitors of Bcl-2 proteins, called BH3-mimetics [17, 18]. These drugs, which mimic the function of the BH3-only proteins, bind to anti-apoptotic Bcl-2 proteins, thereby causing the direct or indirect activation of BAX, BAK, or BOK [19]. The prototypic and broad BH3-mimetic ABT-737 inhibits BCL-2, BCL-W, and BCL-X_L_ and induces cell death in various tumors including HCC, which highly express BCL-X_L_ [20, 21]. Therefore, ABT-737 or the orally available version ABT-263 might be more effective in BCL-X_L_-expressing tumors, e.g., HCC, compared to ABT-199, which more specifically inhibits BCL-2 [22–26].

In addition to therapeutics that target intrinsic apoptosis regulators, anti-cancer drugs activating the extrinsic apoptosis pathway are increasingly coming into focus. Pre-clinical data suggest that TRAIL plays a pivotal role in tumor defense, which is underlined by the observation that TRAIL-deficient mice are more susceptible to chemically induced as well as spontaneous tumor development [27–29]. In contrast to CD95L, TRAIL has been shown to selectively induce apoptosis in transformed, but not in healthy cells, and TRAIL-R1/R2 are highly upregulated in tumors including HCC [30–33]. First-generation TRAIL therapeutics were well tolerated but revealed limited anti-tumor effects, which was mainly attributed to their biologic properties. Therefore, second-generation TRAIL-based molecules with improved anti-tumorigenic activity have been developed and were shown to exhibit an efficient therapeutic response when combined with sensitizing agents, such as small-molecule BCL-X_L_ inhibitors or the proteasome inhibitor bortezomib [34–36].

Using different HCC cell lines and HCC tissues from various patients reflecting HCC heterogeneity, we investigated whether the sorafenib response could be enhanced by the combination with apoptosis-inducing or sensitizing agents, such as TRAIL or the BH3-mimetic ABT-737. Our results reveal that both agents could increase sorafenib-induced cell death, which was dependent on different BH3-only proteins expressed in the HCC cell lines or patient tissues.

## Results

### Sorafenib in combination with TRAIL exerts strong cytotoxic activity in Huh7 but not Hep3B hepatocellular carcinoma cells

**Fig. 1:**
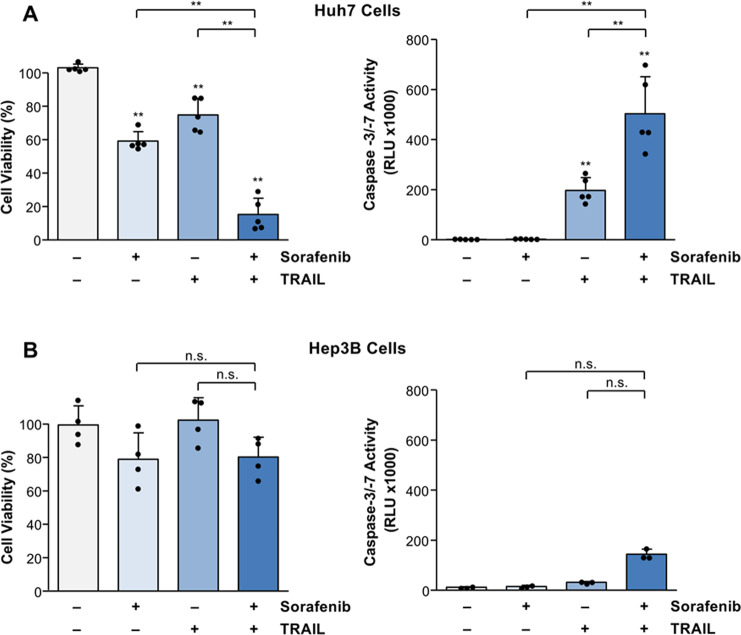
Sorafenib and TRAIL induce cell death and caspase activation in Huh7 cells but not in Hep3B cells. **A** Treatment of Huh7 cells for 8 h with sorafenib (7.5 µg/ml) in combination with TRAIL (50 ng/ml) resulted in a significantly stronger reduction of cell viability (assessed by crystal violet staining) compared to the respective agents alone (left panel). Similarly, treatment with sorafenib and TRAIL significantly enhanced caspase activation as measured by luminometric substrate assay (right panel). **B** Treatment of Hep3B cells with sorafenib or TRAIL alone or with their combination did not result in significant cell death induction (left panel) or caspase activation (right panel). For cell viability results of five (**A**) and four (**B**) independent experiments and for caspase activity results of five (**A**) and three (**B**) independent experiments are shown. *P*-values above the bars refer to control. **p < 0.01; n.s. non-significant.

To unravel the molecular mechanisms of HCC resistance towards sorafenib, we screened several HCC cell lines, which mirror the HCC heterogeneity, for cell death induction by different sorafenib-based anti-cancer drug combinations. We found that Huh7 cells are sensitive to sorafenib- or TRAIL-induced cell death, as indicated by a significant (*p* < 0.01) reduction of cell viability compared to untreated control. Cell viability was further strongly reduced by combined TRAIL and sorafenib treatment compared to the single agents alone (Fig. 1A). The reduction in cell viability was accompanied by a significant (*p* < 0.01) increase of caspase-3/-7 activation, as measured by a luminometric substrate assay [37]. Intriguingly, whereas TRAIL significantly induced caspase activation in Huh7 cells (197.7 ± 50.7 RLU × 1000 vs. 1.7 ± 0.7 RLU × 1000 in untreated control), which was further enhanced (*p* < 0.01) by the combination with sorafenib (503.7 ± 148.2 RLU × 1000), sorafenib alone did not activate caspases (2.6 ± 0.8 RLU × 1000), indicating that apoptosis does not play a major role in sorafenib cytotoxicity (Fig. 1A). In contrast to Huh7 cells, treatment of Hep3B cells with sorafenib or TRAIL alone or their combination did not result in cell death or caspase activation (Fig. 1B). Taken together, we have identified HCC cells sensitive (Huh7) or resistant (Hep3B) to apoptosis induction by sorafenib and TRAIL.

### NOXA determines the sensitivity of HCC cells to TRAIL/sorafenib-induced apoptosis

**Fig. 2:**
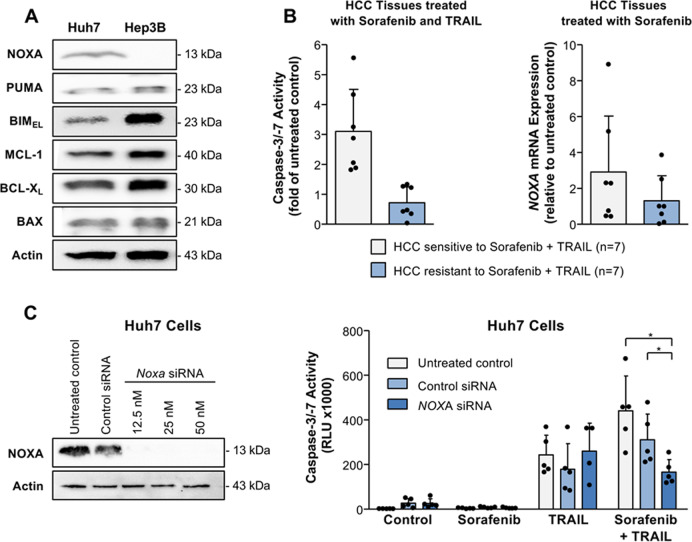
NOXA expression determines apoptosis sensitivity (caspase activation) to sorafenib/TRAIL in Huh7 cells and human HCC tissues. **A** Expression of pro- (NOXA, PUMA, BIM, and BAX) and anti-apoptotic (MCL-1, BCL-X_L_) Bcl-2 proteins in the HCC cell lines Huh7 and Hep3B as detected by Western blotting. In contrast to Huh7 cells, NOXA expression was undetectable in Hep3B cells. **B** HCC tissues from different patients (*n* = 14) were treated for 8 h with sorafenib (7.5 µg/ml) and TRAIL (50 ng/ml; left panel). In contrast to apoptosis-resistant HCC tissues (*n* = 7), in apoptosis-sensitive tissues (*n* = 7) treatment with sorafenib and TRAIL resulted in caspase-3/-7 activation. HCC tissues from the same patients were treated for 8 h with sorafenib (right panel). Sorafenib induced a stronger upregulation of transcripts encoding the BH3-only protein NOXA (assessed by real-time PCR) in HCC tissues sensitive to sorafenib/TRAIL-induced apoptosis (*n* = 7), compared to HCC tissues resistant to this treatment combination (*n* = 7). **C** Effective siRNA-mediated NOXA downregulation in Huh7 cells as determined by Western blotting (left panel). Downregulation of NOXA by siRNA (12.5 nM) did not affect caspase activation in Huh-7 cells treated for 8 h with sorafenib or TRAIL alone. In contrast, caspase activation induced by the combination of sorafenib and TRAIL was significantly reduced in Huh7 cells with *NOXA* downregulation as compared to the respective control cells. The results of five independent experiments are shown. *p < 0.05.

Based on our observation that Huh7 and Hep3B cells reveal a distinct sensitivity towards apoptosis induction by TRAIL and sorafenib, we analyzed the expression pattern of pro- (NOXA, PUMA, BIM, and BAX) and anti-apoptotic (BCL-X_L_, MCL-1) regulators in these HCC cell lines. In contrast to Huh7 cells, NOXA expression was not detected in Hep3B cells. Whereas both cell lines showed similar PUMA and BAX levels, BIM expression was enhanced in Hep3B compared to Huh7 cells. These data therefore suggest that the BH3-only protein BIM could not substitute for NOXA with respect to TRAIL/sorafenib-induced apoptosis. Moreover, Hep3B cells showed a higher expression of the anti-apoptotic regulators BCL-X_L_ and MCL-1 compared to Huh7 cells, which might also account for their apoptosis resistance (Fig. 2A). In addition to Huh7 and Hep3B cells, we analyzed the HCC cell lines HepG2 and HLF and observed similar NOXA, BCL-X_L_, and MCL-1 expression and comparable cell death induction by sorafenib and TRAIL as in Huh7 cells (Supplemental Fig. 1A−C).

We next investigated apoptosis induction by TRAIL and sorafenib in human HCC tissues. For this purpose, we used a previously established liver culture model that allows the culturing and investigation of drug-induced cytotoxicity in intact non-fixed liver tissues [35, 38]. We found that HCC tissues from 50% (*n* = 7) of the patients treated with sorafenib and TRAIL revealed apoptotic cell death, as indicated by an increase of caspase-3/-7 activity (≥1.5 fold compared to the respective untreated control HCC tissue), whereas the other 50% of HCC tissues (*n* = 7) showed apoptosis resistance towards these agents (Fig. 2B, left panel). We then compared HCC tissues either responding or non-responding to sorafenib and TRAIL for the mRNA expression of pro- and anti-apoptotic regulators. Our results showed an upregulation of the mRNA of the pro-apoptotic regulator NOXA (Fig. 2B, right panel) but not of other pro-apoptotic members of the Bcl-2 family, e.g., BIM, PUMA, BAX, or BAD (Supplemental Fig. 2), in sorafenib-treated HCC tissues, which responded to sorafenib and TRAIL, as compared to HCC tissues resistant to this treatment combination. These data therefore suggest that NOXA is induced by sorafenib and plays an important role for TRAIL-mediated apoptosis sensitivity.

To further unravel the role of NOXA in sorafenib/TRAIL-induced apoptosis, we performed siRNA-mediated *NOXA*-knockdown in Huh7 cells (Fig. 2C, left panel) and analyzed caspase activation induced by this drug combination. We found that *NOXA*-knockdown significantly (*p* < 0.05) reduced caspase-3/-7 activity in Huh7 cells treated with TRAIL and sorafenib (166.4 ± 56.3 RLU × 1000) compared to untreated (440.8 ± 156.1 RLU × 1000) or control-siRNA-transfected (311.1 ± 115.3 RLU × 1000) cells (Fig. 2C, right panel). These data indicate that NOXA plays an important role for apoptosis induction in HCC cells by TRAIL and sorafenib.

### The BH3-mimetic ABT-737 enhances sorafenib-mediated cell death in NOXA-deficient HCC cells

**Fig. 3:**
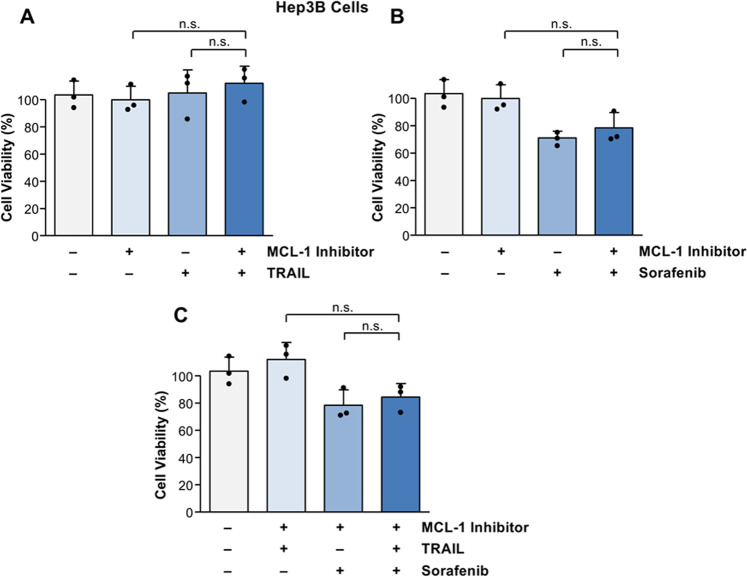
MCL-1 inhibition does not sensitize for cell death induction by sorafenib and TRAIL in Hep3B cells. **A** No significant cell death was observed in Hep3B cells treated with TRAIL (50 ng/ml) or the MCL-1 inhibitor A-1210477 (10 µM) alone. Combined treatment of Hep3B cells with TRAIL and the MCL-1 inhibitor did not result in enhanced cell death. **B** Sorafenib alone or in combination with the MCL-1 inhibitor did not significantly reduce cell viability in Hep3B cells. **C** Hep3B cells are not sensitized for cell death by combined treatment of sorafenib and TRAIL, both in the presence or absence of the MCL-1 inhibitor. The figures represent the results of three independent experiments. N.s. non-significant.

NOXA can be inhibited by its anti-apoptotic binding partner MCL-1. Since enhanced expression of MCL-1 might be involved in the decreased apoptosis sensitivity of Hep3B cells, we asked whether treatment with the MCL-1 inhibitor A-1210477 sensitizes to TRAIL/sorafenib-induced cell death. However, Hep3B cells were neither sensitized for TRAIL- nor for sorafenib-mediated cell death by MCL-1 inhibition (Fig. 3A, B). MCL-1 inhibition did also not sensitize Hep3B cells for cell death induction by combined TRAIL and sorafenib treatment (Fig. 3C). Therefore, inhibition of MCL-1 is apparently not an efficient therapeutic strategy in NOXA-deficient HCC.

**Fig. 4:**
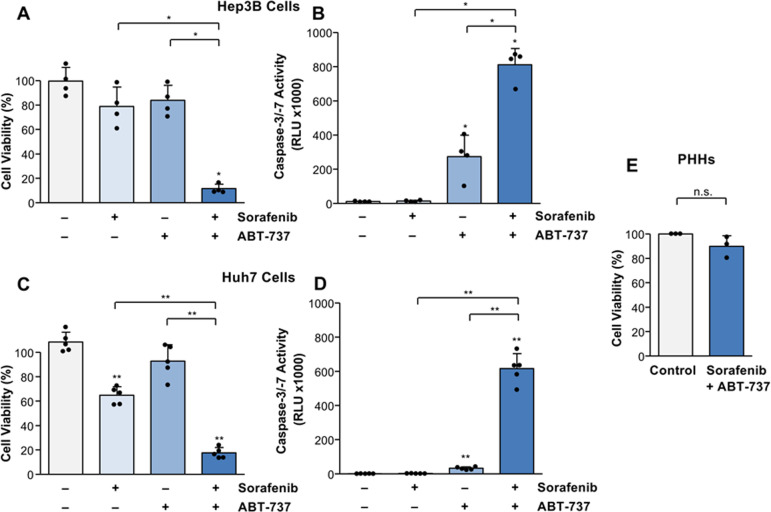
The BH3-mimetic ABT-737 sensitizes Huh7 and Hep3B cells but not primary human hepatocytes (PHHs) for sorafenib-mediated cell death and caspase activation. **A** Treatment of Hep3B cells for 8 h with sorafenib (7.5 µg/ml) or ABT-737 (5 µM) alone did not significantly reduce cell viability in Hep3B cells, whereas their combination strongly increased cell death. **B** Compared to the respective agents alone, combined treatment (8 h) of sorafenib and ABT-737 increased caspase-3/-7 activation in Hep3B cells. **C** In contrast to Hep3B cells, sorafenib significantly reduced cell viability in Huh7 cells, which was even more pronounced by the combination with ABT-737. **D** Whereas no caspase activation was observed by sorafenib treatment alone, its combination with ABT-737 significantly induced caspase-3/-7 activation in Huh7 cells. **E** Compared to untreated cells, treatment of PHHs (obtained from three different donors) for 8 h with sorafenib and ABT-737 did not induce cell death. Results of four (**A**, **B**) and five (**C**, **D**) independent experiments are shown. Significances above the bars refer to control. *p < 0.05; **p < 0.01; n.s. non-significant.

We next investigated the effect of the BH3-mimetic ABT-737, which targets anti-apoptotic BCL-2 and BCL-X_L_ in Hep3B cells. Whereas ABT-737 or sorafenib alone did not reduce cell viability, cell death could be significantly (*p* < 0.05) increased by co-treatment with both drugs (Fig. 4A). Enhanced cell death was associated with a significant (*p* < 0.05) increase of caspase activation in Hep3B cells treated with sorafenib in combination with ABT-737 (812.0 ± 95.4 RLU × 1000) compared to the single agents sorafenib (13.9 ± 4.7 RLU × 1000) or ABT-737 (273.6 ± 126.1 RLU × 1000) and to untreated control (11.4 ± 2.4 RLU × 1000). Thus, this treatment combination was able to efficiently induce apoptosis in NOXA-deficient HCC cells (Fig. 4B).

Since BCL-X_L_ is frequently upregulated in HCC [20] and we found a stronger expression in Hep3B compared to Huh7 cells (Fig. 2A), we compared the efficacy of sorafenib combined with ABT-737 also in Huh7 cells. Similar to Hep3B cells, the combination of sorafenib and ABT-737 significantly (*p* < 0.01) reduced cell viability in Huh7 cells compared to the respective agents alone (Fig. 4C). Cell death induction by the combination of sorafenib and ABT-737 was associated with a strong increase of caspase activity in Huh7 cells (616.0 ± 87.6 RLU × 1000) compared to sorafenib (2.6 ± 0.8 RLU × 1000) or ABT-737 (32.9 ± 8.2 RLU × 1000) alone or to untreated control (1.7 ± 0.7 RLU × 1000; Fig. 4D). Thus, the combination of sorafenib and ABT-737 resulted in significant caspase activation and cell death induction in both NOXA-deficient (Hep3B) and -expressing (Huh7) HCC cells. Furthermore, we did not find a significant induction of cell death by sorafenib and ABT-737 in non-transformed primary human hepatocytes (Fig. 4E).

### ABT-737 does not restore TRAIL sensitivity in NOXA-deficient HCC cells

**Fig. 5:**
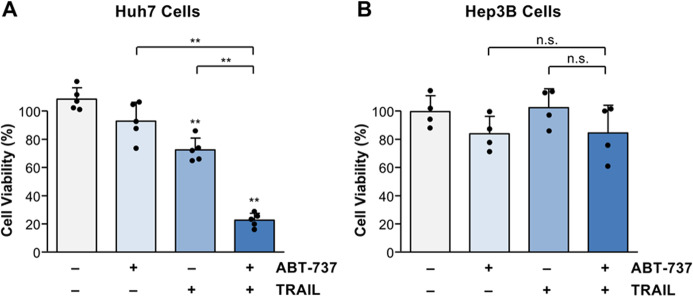
ABT-737 sensitizes for TRAIL-induced cell death in Huh7 but not Hep3B cells. **A** Detection of cell viability in Huh7 cells treated with TRAIL (50 ng/ml) or ABT-737 (5 µM) alone or in combination for 8 h. TRAIL-mediated cell death was significantly increased by combination with ABT-737 in Huh-7 cells. **B** Neither TRAIL alone nor its combination with the BH3-mimetic reduced cell viability in Hep3B cells. Results of five (**A**) or four (**B**) independent experiments are shown. Significances indicated above the bars refer to control. **p < 0.01; n.s. non-significant.

As ABT-737 sensitizes Hep3B cells to sorafenib-mediated cell death, we asked whether Bcl-2 inhibition also restores TRAIL sensitivity. In contrast to Huh7 cells, in which TRAIL combined with ABT-737 strongly increased cell death compared to the respective agents alone (*p* < 0.01; Fig. 5A), this drug combination failed to enhance cell death in Hep3B cells (Fig. 5B). These data suggest that NOXA is required for TRAIL-based treatment combinations and ABT-737 could therefore not restore TRAIL sensitivity in NOXA-deficient Hep3B cells.

### ABT-737 does not enhance sorafenib sensitivity in BIM-deficient HCC cells

**Fig. 6:**
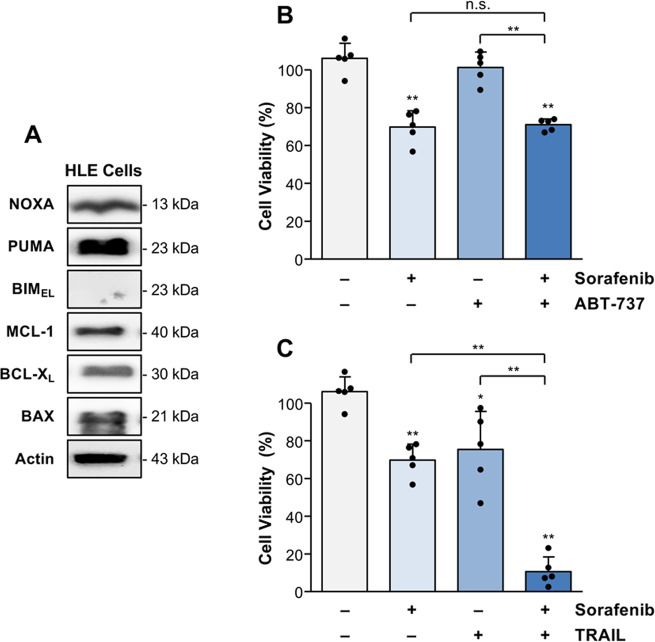
TRAIL but not ABT-737 enhances sorafenib-mediated cell death in BIM-deficient HLE cells. **A** Western blot analysis of the expression of pro-apoptotic (NOXA, PUMA, BIM, and BAX) and anti-apoptotic Bcl-2 molecules (MCL-1, BCL-X_L_) in HLE cells revealed a lack of BIM expression. **B** Viability of HLE cells treated for 8 h with sorafenib (7.5 µg/ml) or ABT-737 (5 µM) alone or in combination. Compared to sorafenib alone, HLE cell death was not enhanced by the combination with ABT-737. **C** In contrast, cell death could be strongly enhanced in HLE cells by the combination of sorafenib with TRAIL (50 ng/ml). Results of five (**B**, **C**) independent experiments are shown. Western blot analysis was performed on the same membrane as shown in Fig. 2. Significances indicated above the bars refer to control. *p < 0.05; **p < 0.01; n.s. non-significant.

Hep3B cells are BIM-proficient and can be sensitized to sorafenib- but not TRAIL-induced cell death by ABT-737. We therefore evaluated the role of BIM for sorafenib sensitivity of HCC cells. We found that the HCC cell line HLE lacks BIM expression but reveals strong expression of other BH3-only proteins, such as PUMA and NOXA (Fig. 6A). Similar to Huh7 and Hep3B cells, HLE cells express the pro-apoptotic Bcl-2 molecule BAX and the anti-apoptotic regulators MCL-1 and BCL-X_L_. Treatment of HLE cells with sorafenib and ABT-737 did not further reduce cell viability, indicating that the sorafenib response cannot be increased by ABT-737 in the absence of BIM (Fig. 6B). In contrast to ABT-737, sorafenib cytotoxicity was strongly (*p* < 0.01) increased by co-treatment with TRAIL (Fig. 6C), again suggesting that TRAIL-mediated cell death sensitization mainly depends on NOXA expression.

### Mechanisms of sorafenib-mediated reduction of HCC cell viability

**Fig. 7:**
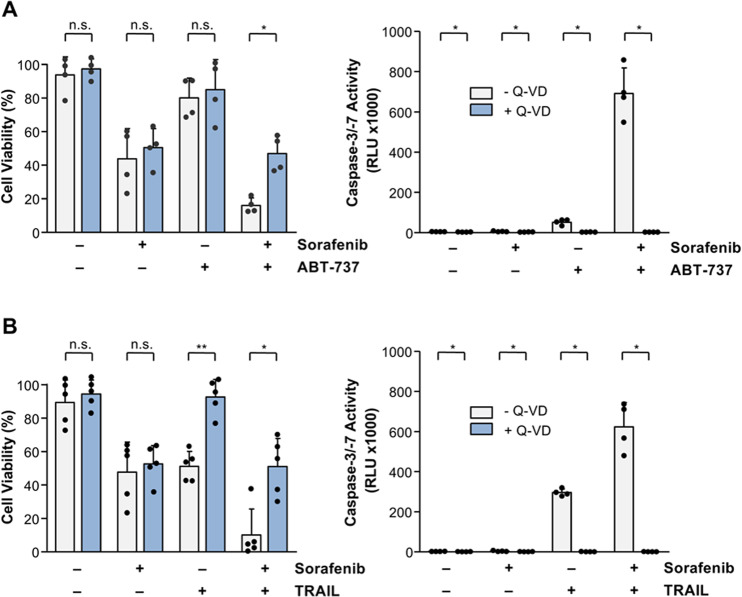
Sorafenib-induced cell death in Huh7 cells is mainly caspase-independent. **A** Analyses of cell death and caspase activation in Huh-7 cells treated for 8 h with sorafenib or ABT-737 alone or in combination in the presence or absence of the caspase inhibitor Q-VD (10 µM). Caspase activation induced by the combination of sorafenib and ABT-737 was completely inhibited by pre-incubation (1 h) of Huh7 cells with Q-VD, whereas cell death induced by this combination could only be inhibited to the level of sorafenib-mediated reduction of cell viability. **B** Similarly, caspase activation induced by TRAIL or its combination with sorafenib was almost completely inhibited by pre-treatment with Q-VD for 1 h. However, cell death induced by sorafenib and TRAIL was inhibited by Q-VD only to the level of sorafenib-mediated cell death. Results of four (**A**) and five (**B**) independent experiments are shown for cell viability and results of four independent experiments are shown for caspase activation (**A**, **B**). *p < 0.05; **p < 0.01; n.s. non-significant.

To further explore the cell death mode induced by sorafenib in combination with ABT-737 or TRAIL, we pre-incubated Huh7 cells with the pan-caspase inhibitor Q-VD before treatment with the different drug combinations. Caspase-3/-7 activation could be completely inhibited by co-treatment with Q-VD and sorafenib in the combination of ABT-737 (0.6 ± 0.1 RLU × 1000 vs. 691.6 ± 127.4 RLU × 1000; Fig. 7A) or TRAIL (0.9 ± 0.3 RLU × 1000 vs. 623.6 ± 121.6 RLU × 1000; Fig. 7B). However, reduction of cell viability induced by sorafenib alone was not affected by caspase inhibition (Fig. 7A, B). These data suggest that sorafenib as single-agent primarily induces non-programmed cell death, but can potentiate apoptotic cell death in combination with BH3-mimetics or TRAIL, depending on the individual expression profile of BH3-only proteins.

**Fig. 8:**
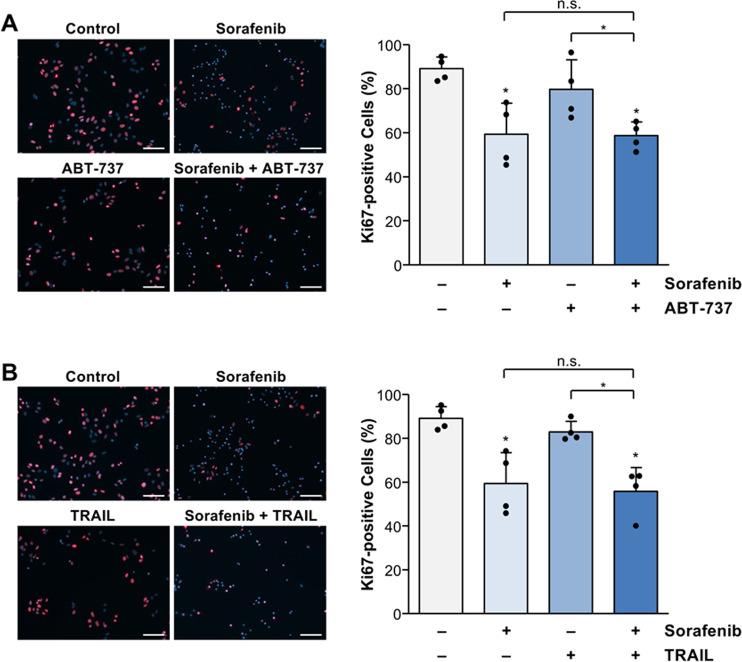
Sorafenib reduces cell proliferation in Huh7 cells, which is not affected by the combination with TRAIL or ABT-737. **A** Analyses of proliferation inhibition by immunohistochemical assessment of Ki67-positive (red) Huh7 cells treated for 8 h with sorafenib (7.5 µg/ml) and/or ABT-737 (5 µM). Sorafenib but not ABT-737 alone significantly reduced cell proliferation. The combination of sorafenib and ABT-737 did not further increase inhibition of proliferation as compared to sorafenib alone. **B** TRAIL treatment (50 ng/ml) of Huh7 cells did not significantly affect cell proliferation. The combined treatment of Huh7 cells with sorafenib and TRAIL for 8 h did not further inhibit cell proliferation compared to sorafenib alone. Results of four (**A**, **B**) independent experiments are shown. Significances indicated above the bars refer to control. *p < 0.05; n.s. non-significant; scale bars = 100 µm.

We additionally investigated a potential anti-proliferative efficacy of sorafenib in combination with ABT-737 or TRAIL in Huh7 cells by Ki67 staining. In contrast to ABT-737 (Fig. 8A) or TRAIL (Fig. 8B), sorafenib treatment resulted in a significant (*p* < 0.05) reduction of Huh7 cell proliferation (59.3 ± 14.1% Ki67-positive cells) compared to untreated control (89.1 ± 5.4% Ki67-positive cells) which, however, was not significantly affected by co-treatment with ABT-737 (58.7 ± 6.2% Ki67-positive cells; Fig. 8A) or TRAIL (55.8 ± 10.8% Ki67-positive cells; Fig. 8B).

## Discussion

To explore the molecular mechanisms of HCC resistance towards sorafenib-based treatment, we have screened several HCC cell lines, which mirror the HCC heterogeneity, for apoptosis induction by different anti-cancer drugs. We found that Huh7 cells were sensitive to sorafenib-induced cell death, which was strongly enhanced by the combination with TRAIL. In contrast, Hep3B cells revealed apoptosis resistance to sorafenib and TRAIL. Based on the distinct drug sensitivity, we compared the expression of pro- and anti-apoptotic Bcl-2 molecules in HCC cells. We found that, unlike drug-sensitive Huh-7 cells, Hep3B cells lacked the expression of NOXA, indicating that NOXA could be involved in the resistance of HCC.

To unravel the role of NOXA for sorafenib and TRAIL-induced apoptosis, we performed siRNA-mediated *NOXA*-knockdown in Huh7 cells, which significantly impaired caspase activation by this drug combination. Similarly, we found enhanced *NOXA* expression in sorafenib-treated HCC tissues responding to apoptosis induction by combined treatment with TRAIL compared to non-responding HCC tissues. *NOXA* is transcriptionally regulated by the tumor suppressor p53, which is mutated in up to 30% of HCC cases [6, 39–41]. Whereas Huh7 cells reveal p53 point mutations without functional loss, Hep3B cells show chromosomal deletion of the p53 gene locus which might account for the lack of NOXA expression and TRAIL resistance [42, 43]. This is in line with a recent study demonstrating that p53-deficient colorectal or lung carcinoma cells are less sensitive to TRAIL-mediated cell death compared to respective wild-type cells [44].

Based on our observation that Hep3B cells reveal enhanced expression of MCL-1, which antagonizes NOXA, we asked whether treatment response could be restored by the MCL-1 inhibitor A-1210477. NOXA-deficient Hep3B cells treated with the MCL-1 inhibitor were, however, not sensitized for cell death induction by sorafenib and/or TRAIL. In contrast to MCL-1 inhibition, treatment of Hep3B cells with ABT-737 and sorafenib significantly induced cell death and caspase activation to a similar extent as observed in Huh7 cells. Thus, sorafenib sensitivity could be restored in Hep3B cells by the Bcl-2 inhibitor ABT-737, which inhibits BCL-2, BCL-X_L_, and BCL-W but not MCL-1. It is interesting to note that, unlike ABT-737, ABT-199 that primarily targets BCL-2 was unable to induce efficient cell death in combination with sorafenib or regorafenib in liver cancer cells [24, 45], indicating that targeting of BCL-X_L_, e.g., by ABT-737, might be more effective in HCC.

ABT-737 has been previously reported to cause severe toxicity in *Mcl1*-deficient mice [46]. Since sorafenib can also downregulate MCL-1 [24], we analyzed the potential toxicity of combined treatment of sorafenib and ABT-737 in non-transformed, i.e., primary human hepatocytes (PHH). We did not find a significant induction of cell death in PHHs by this drug combination. This is in line with previous studies, demonstrating that sorafenib treatment of Bcl-x_L_-deficient mice showed no increased liver injury and that combined application with BH3-mimetics in leukemia or HCC xenograft mice was well tolerated and resulted in only mild thrombocytopenia [46–48].

Previous studies showed that ABT-737 or its orally active version ABT-263 also sensitizes human cancer cells to TRAIL-induced apoptosis without exerting toxicity in normal liver cells [49, 50]. We therefore analyzed the combination of ABT-737 with TRAIL in Huh7 and Hep3B cells for their ability to induce cell death. TRAIL-induced apoptosis was significantly increased in the presence of ABT-737 in Huh7 cells. However, TRAIL sensitivity could not be restored in NOXA-deficient Hep3B cells despite the high expression of BIM. Since previous phase-2 trials evaluating sorafenib in combination with TRAIL agonists did not meet the primary efficacy end point [51, 52], it will be interesting to explore whether subgroups of patients with different expression of BH3-only proteins, such as NOXA, reveal a different treatment response to this drug combination. Further studies re-evaluating the expression levels of BH3-only proteins in HCC patients treated with TRAIL-based therapies are therefore required.

Evidence exists that also BIM plays a role for TKI response. We found no BIM protein expression in HLE cells and therefore analyzed this HCC cell line for its drug response. In contrast to BIM-expressing HCC cells, including Huh7, Hep3B, HepG2, and HLF, BIM-deficient HLE cells were not sensitized to sorafenib by ABT-737. A similar observation was made in leukemia cells with siRNA-mediated *BIM*-knockdown, which revealed resistance to combined treatment with a MEK inhibitor and ABT-737 or sorafenib and obatoclax [48, 53]. A role of BIM for sorafenib response is further underlined by the observation that MEK inhibition leads to an upregulation of BIM in HCC xenografts concomitant with increased sorafenib sensitivity [54].

The observation that ABT-737 does not sensitize for sorafenib-induced cell death in the absence of BIM protein expression could be explained by the fact that BH3-mimetics cause the release of BIM from BCL-2 and BCL-X_L_, which is required for BAX/BAK activation and apoptosis induction [19, 55, 56]. This is in line with a previous study revealing that BIM determines ABT-737 sensitivity of small cell lung cancer cells with low NOXA expression [57] and with our data that ABT-737 in combination with sorafenib induces cell death in Hep3B cells that lack NOXA but reveal BIM expression. Accordingly, a recent study found a compensatory relationship between NOXA and BIM for the sensitization of ovarian carcinoma cells to ABT-737-based treatments [58]. Nevertheless, it was demonstrated that in the absence of NOXA, BIM was sequestered by MCL-1, which could be prevented by NOXA and its binding to MCL-1 [55, 59, 60]. We demonstrate, however, that BIM-deficient HLE cells are sensitive to sorafenib in combination with TRAIL, presumably due to their high NOXA and PUMA expression. Response to a certain sorafenib-based drug combination might therefore depend on the tumor-specific BH3-only protein expression.

To further explore the mode of cell death induced by sorafenib in combination with ABT-737 or TRAIL, we investigated the effect of the caspase inhibitor Q-VD on the cytotoxicity of the different drug combinations. Caspase activation could be completely inhibited by Q-VD following treatment of Huh7 cells with ABT-737 or TRAIL. However, cell death induced by sorafenib alone was not prevented by caspase inhibition. To further unravel the mode of cell death, we analyzed a contribution of necroptosis or ferroptosis by pre-incubation of Huh7 cells with necrostatin and deferoxamine, respectively, before cytotoxic drug treatment. However, we found no evidence for a contribution of necroptosis or ferroptosis in cell death induced by sorafenib, either alone or in combination with ABT-737 or TRAIL (data not shown). These data suggest that sorafenib as single agent primarily induces non-programmed cell death but can potentiate apoptotic cell death in combination with BH3-mimetics or TRAIL, depending on the individual expression profile of BH3-only proteins.

As mentioned above, mutation and thus activation of survival, e.g., the Wnt/β-catenin pathway, which can be observed in ~40% of HCC, results in enhanced tumor cell proliferation and invasion [6, 61, 62]. In addition to cell death induction, sorafenib has also anti-proliferative capacity, e.g., by inhibition of the MAP kinase or Akt/mTOR signaling. Furthermore, sorafenib downregulates cell cycle regulators, such as cyclin D1/D2, thereby inducing cell cycle arrest [63–66]. We found that sorafenib inhibits cell proliferation, which was not further potentiated in combination with TRAIL or ABT-737. A previous study in a leukemia xenograft mouse model revealed that the combined treatment with sorafenib and BH3-mimetics results in a marked reduction of tumor growth and significantly prolonged survival, which was associated with apoptosis induction [48]. Thus, for sorafenib-based treatment combinations induction of tumor cell death rather than inhibition of proliferation might mainly determine the therapeutic efficacy and clinical outcome. Apoptosis sensitivity of HCC to sorafenib-based combinations depends on the expression pattern of BH3-only proteins. The molecular profiling of BH3-only proteins could therefore be helpful for patient stratification with respect to individualized therapeutic strategies in HCC.

## Material and methods

### Culture of Huh7, Hep3B, HLE, HepG2, HLF, primary human hepatocytes, and HCC tissues

Huh7, Hep3B, HLE, HepG2, and HLF cells were cultured in Dulbecco’s modified Eagle’s medium (Thermo Fisher Scientific, Waltham, MA, USA) supplemented with 1 g/L glucose, 10% fetal calf serum (Biochrom AG, Berlin, Germany) and 1% penicillin/streptomycin (Merck Millipore, Burlington, MA, USA). PHHs were isolated as described [67] from liver tissues of different donors (*n* = 3) undergoing partial hepatectomy. PHHs were cultured for 36 h in William’s medium E GlutaMAX (Thermo Fisher Scientific), supplemented with 1% penicillin/streptomycin, 10% fetal calf serum, and 100 nM dexamethasone for the first 12 h. Freshly isolated HCC explants (*n* = 14) were precisely cut into 125 mm^3^ cubes and incubated in 24-well plates with modified Eagle’s medium (Invitrogen, Carlsbad, CA, USA) supplemented with 1% human serum, 4 U/mL human insulin, 20 mM HEPES, 2 mM L-glutamine, 0.2 g/L MgCl_2_ × 6 H_2_O, 1 × vitamin solution, 20 mg/L L-ornithine-HCl, 50 mg/L ascorbic acid, 50 µg/mL gentamycin and 8 µg/ml dexamethasone [35].

The following drugs and concentrations were used for the treatment (8 h) of HCC cells, PHHs or human HCC tissues: 7.5 µg/ml sorafenib (Selleckchem, Houston, TX, USA), 50 ng/ml TRAIL (Enzo Life Sciences, Lörrach, Germany), 5 µM ABT-737 (Selleckchem), 10 µM MCL-1 inhibitor A-1210477 (Selleckchem). Huh7 cells were pre-treated for 1 h with 10 µM Q-VD (R&D, Minneapolis, USA) before adding sorafenib in the absence or presence of TRAIL or ABT-737 (8 h). As control, hepatocytes were treated with 0.3% DMSO (Sigma-Aldrich, St. Louis, MO, USA) or left untreated.

In the knockdown experiments, Huh7 cells were transfected for 48 h with 12.5 nM *Noxa*-targeting or non-targeting control siRNA (Dharmacon, Lafayette, LA, USA) by using lipofectamine RNAiMAX reagent (Thermo Fisher Scientific). Huh7 cells incubated with Opti-MEM medium (Gibco, Thermo Fisher Scientific) served as untreated control.

### Measurement of cell viability and caspase activation

For detection of cell viability, crystal violet staining was performed [68]. The absorbance was measured at 590 nm. Values of untreated cells represented 100% viability. Caspase-3/-7 activation was measured in triplicates by a luminescent substrate assay (Caspase-Glo, Promega, Mannheim, Germany) [69]. Cell extracts were diluted in 50 mM Tris-HCl pH 7.4, 10 mM KCl and 5% glycerol to reach a final protein concentration of 0.1 mg/ml. Then, 10 µL of the extracts were incubated with 10 µL of the caspase substrate DEVD-luciferin and luciferase reagent for 2 h at room temperature. Luminescence was measured in relative light units (RLU) by a luminometer (LB 960, Berthold Technologies, Bad Wildbad, Germany).

### Immunoblotting

Immunoblotting was performed essentially as described [70]. Cell lysates were separated under reducing conditions on 15% SDS-polyacrylamide gels and electroblotted to a polyvinylidene difluoride membrane. After 1 h of blocking in 5% non-fat dry milk powder in TBST, membranes were incubated with primary antibodies overnight at 4°C. Polyclonal anti-actin, polyclonal anti-BCL-X_L_, and monoclonal anti-NOXA antibodies were from Santa Cruz (Santa Cruz Biotechnology, Dallas, TX, USA). Polyclonal anti-BAX was purchased from Merck-Millipore (Darmstadt, Germany), monoclonal anti-BIM from Enzo Life Sciences, monoclonal anti-MCL-1 from Cell Signaling (Danvers, MA, USA), and polyclonal anti-PUMA antibody from Abcam (Cambridge, UK).

### RNA preparation and quantitative real-time PCR

RNA was prepared from liver tissues using the AllPrep DNA/RNA/Protein Mini Kit (Qiagen, Hilden, Germany) according to the manufacturer’s protocol. RNA was reverse-transcribed using the QuantiTect Reverse Transcription kit (Qiagen). Quantitative RT-PCR was performed in triplicates employing a SYBR Green PCR Master Mix (Thermo Fisher Scientific). QuantiTect Primer Assays (Qiagen) were used for the detection of *NOXA*, *PUMA*, *BIM*, *BAX*, *BAD,* and *GAPDH* as reference gene. Data were analyzed using the comparative (ΔΔ*C*_*T*_) method with normalization to GAPDH expression.

### Ki67 staining

Huh7 cells were seeded on poly-L-lysine (Sigma-Aldrich)-coated coverslips and incubated with the different drugs. Cells were then fixed with 4% paraformaldehyde and incubated for 2 min in a buffer containing 0.1% Triton-X-100 and 0.1% trisodium citrate. After repeated washings, nonspecific binding was blocked with 2% bovine serum albumin and 5% goat serum in PBS for 10 min. Cells were then stained with anti-Ki67 antibody (Dako, Santa Clara, CA, USA) for 1 h at room temperature as described [71]. After repeated washings with PBS, cells were incubated with a Cy3-labeled secondary antibody (Jackson Laboratory, Bar Harbor, ME, USA) for an additional hour. For nuclear staining, DAPI (4′,6-diamidino-2-phenylindole) was used (Thermo Fisher Scientific). The percentage of Ki67-positive cells was assessed by analyzing four microscopic fields at 200× magnification.

### Statistical analyses

For statistical analyses, Mann-Whitney’s U test (non-equal data distribution) or one-sample *t* test (treated compared to untreated PHHs) was performed using GraphPad Prism 5.0 (GraphPad Software, San Diego, CA, USA). Data represent mean ± standard deviation. The results are shown as bars with individual data points. A *p*-value of less than 0.05 was considered significant.

## Supplementary information

Supplemental Material
