# Supplementary material for: BH3-only protein expression determines hepatocellular carcinoma response to sorafenib-based treatment
Source: Cell Death Dis. 2021 Jul 26;12(8):736. doi: 10.1038/s41419-021-04020-z (PMC8313681; doi:10.1038/s41419-021-04020-z)
Supplement: Supplementary file 1 — Supplemental Material [file 41419_2021_4020_MOESM1_ESM.docx]

**Supplemental Figure 1**


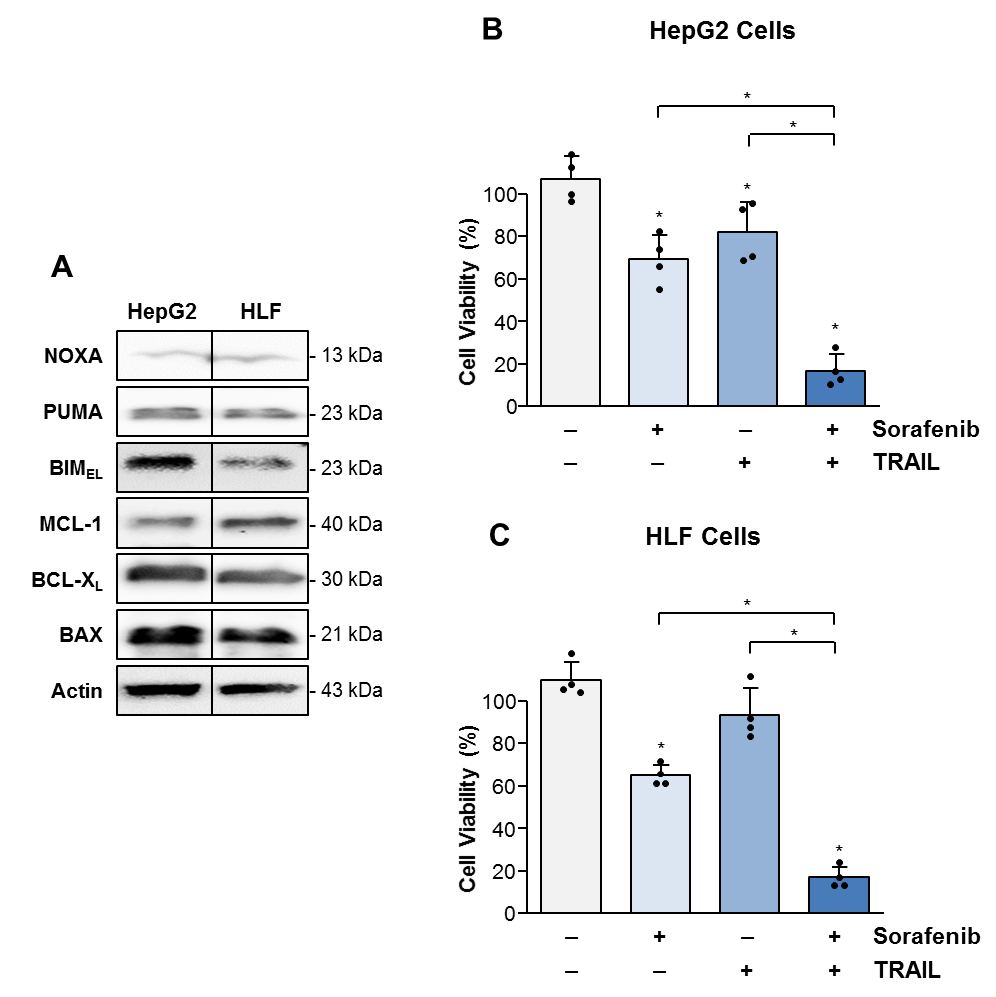


**Supplemental Figure 1. Sorafenib and TRAIL induce cell death in NOXA-expressing HepG2 and HLF cells. A** Western blot analysis of the expression of pro-apoptotic (NOXA, PUMA, BIM, BAX) and anti-apoptotic Bcl-2 molecules (MCL-1, BCL-X_L_) in HepG2 and HLF cells. A similar NOXA expression pattern was observed in HepG2 and HLF compared to Huh7 cells. **B** Treatment of HepG2 cells for 8 h with sorafenib (7.5 µg/ml) in combination with TRAIL (50 ng/ml) resulted in a significantly stronger reduction of cell viability compared to the respective agents alone. **C** Similarly, in HLF cells cell death induction was significantly increased by the combined treatment with sorafenib and TRAIL compared to the single drugs alone. Results of 4 (B, C) independent experiments are shown. Western blot analysis was performed on the same membrane as shown in figure 2 and 6. The vertical lines indicate juxtaposition of non-adjacent lanes from the same blot. Significances above the bars refer to control. *p<0.05.

**Supplemental Figure 2**

**
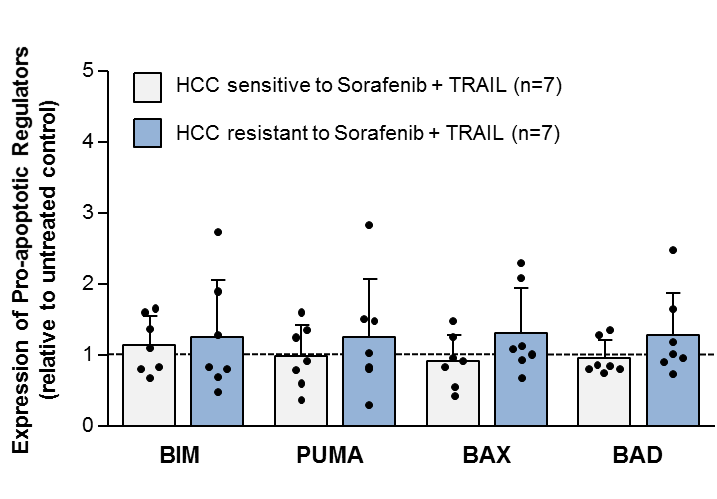
**

**Supplemental Figure 2. Expression of pro-apoptotic regulators in sorafenib-treated human HCC tissues**. In contrast to *NOXA* mRNA expression (Figure 2), no up-regulation of pro-apoptotic *BIM, PUMA, BAX* or *BAD* (assessed by real-time PCR) could be observed in sorafenib-treated HCC tissues, which were sensitive to sorafenib/TRAIL-induced apoptosis (n=7) compared to HCC tissues with resistance to this treatment combination (n=7). The same HCC tissues were analyzed as in Figure 2.
